# Real-time wash-free detection of unlabeled PNA-DNA hybridization using discrete FET sensor

**DOI:** 10.1038/s41598-017-16028-7

**Published:** 2017-11-16

**Authors:** Matti Kaisti, Anssi Kerko, Eero Aarikka, Petri Saviranta, Zhanna Boeva, Tero Soukka, Ari Lehmusvuori

**Affiliations:** 1 University of Turku, Department of Future Technologies, 20500 Turku, Finland; 2 University of Turku, Department of Biotechnology, 20520 Turku, Finland; 3 Åbo Akademi University, Department of Science and Engineering, 20500 Turku, Finland; 4 Medical Biotechnology Centre, VTT Technical Research Centre of Finland, Espoo FI-02044 VTT, Finland

## Abstract

We demonstrate an electrochemical sensor for detection of unlabeled single-stranded DNA using peptide nucleic acid (PNA) probes coupled to the field-effect transistor (FET) gate. The label-free detection relies on the intrinsic charge of the DNA backbone. Similar detection schemes have mainly concentrated on sensitivity improvement with an emphasis on new sensor structures. Our approach focuses on using an extended-gate that separates the FET and the sensing electrode yielding a simple and mass fabricable device. We used PNA probes for efficient hybridization in low salt conditions that is required to avoid the counter ion screening. As a result, significant part of the target DNA lies within the screening length of the sensor. With this, we achieved a wash-free detection where  typical gate potential shifts are more than 70 mV with 1 µM target DNA. We routinely obtained a real-time, label- and wash-free specific detection of target DNA in nanomolar concentration with low-cost electronics and the responses were achieved within minutes after introducing targets to the solution. Furthermore, the results suggest that the sensor performance is limited by specificity rather than by sensitivity and using low-cost electronics does not limit the sensor performance in the presented sensor configuration.

## Introduction

Molecular diagnostics has become a remarkable market segment valued approximately at $5 billion. The first nucleic acid based assay was approved 1986 by the United States Food and Drug Administration (FDA) and nowadays there is more than 220 FDA-approved or cleared molecular diagnostic tests^1^. In recent years, there has been increasing interest for point-of-care (POC) molecular diagnostic tests that could be performed outside laboratories at the site where the patient is located. Rapid POC tests would be valuable especially for the detection of infectious diseases in resource poor settings, for example, the early identification of Tuberculosis would improve patient recovery and prevent the spread of the disease. POC tests needs to be portable, easy-to-use eliminating the requirement for trained personnel, inexpensive especially in less-developed countries, and also specific and sensitive with very few false-positive and false-negative results^2^.

The existing molecular diagnostic systems are mainly based on optical detection with fluorescent labels. Fluorescence-based wash-free optical methods are well-known and widely used, and the fluorometric detection techniques have sufficient sensitivity^3^. However, the optical detection methods require excitation light source and light detector that transforms fluorescence light into measurable electrical signal, and also optical connection to the reaction tube. Therefore, the optical systems are often relatively large and expensive to be used in hand-held POC devices.

In recent years, optics-free electrochemical biosensors have raised significant attention. However, at the moment, there are only few electrochemical bionsensor based molecular diagnostic tests commercially available for diagnostic purposes, such as, Atlas Genetics io System^4^ and GenMark Diagnostics eSensor^5^ based system. These two tests include polymerase chain reaction (PCR) based target nucleic acid amplification and amperometric detection systems utilizing ferrocene labeled oligonucleotide probes. In both methods, the DNA detection is performed after the PCR in a microfluidic channel and the detection additionally contains a washing step.

Label-free electrochemical DNA detection methods based on field-effect transistor (FET) provide significant promise due to their scalability and they can be constructed by tethering the recognition molecules directly to the sensor’s sensing surface. This type of biosensor is small-size and can be created using standard low-cost electronics. The basic principle of the detection relies on a probe DNA attached to the FET gate that specifically hybridizes to the target DNA. Since the DNA backbone has intrinsic negative charge, the change in surface charge conditions lead to a change in the FET channel conductance, which can be measured using electrochemical cell consisting of a FET gate as the working electrode together with a reference electrode immersed into a solution. This basic detection scheme has been described earlier^6–12^ although the details and results vary greatly due to the vast amount of different electrical structures, surface materials and immobilization chemistries.

One of the well-known difficulties in the detection of large polyelectrolyte molecules such as DNA is due to the counter-ions that effectively screen the intrinsic charge of macromolecules^13,14^. This effect is significant at mM range of salt concentrations that are required for efficient DNA-DNA hybridization. In order to avoid the screening effect, a change of a reaction buffer from high ionic strength in hybridization to low ionic strength in detection phase is used for enhanced signal responses^15–18^. This adds complexity, measurement time, and cost to the sensing system and makes it more difficult to miniaturize. Additionally, the reliability of the results is compromised as in many cases the change of salt concentration also changes the recorded output. New methods to circumvent this problem include using a high frequency excitation that breaks-up the double layer^19^ as well as using a polymer surface modification that increases the effective Debye length^20^. However, these methods require either complex measurement equipment or additional functionalization steps^12^.

DNA detection sensitivity in nanomolar range has been achieved with a wash- and label-free sensor^7^. The detection was based on measuring capacitive currents using a lock-in-amplifier, which significantly adds cost and complexity if *in-situ* applications are considered. The use of diamond as a gate material for FET has been used for a real-time wash free detection of DNA 21-mer oligonucleotide hybridization without using any labels with good sensitivity and spesificity^21^ The benefit of system is described to originate from favorable capacitive gate properties. Another DNA detection study described the use of peptide nucleic acid probe immobilized on graphene oxide with high sensitivity and specificity^22^. However, the detection was achieved without real-time measurement using a 1 hour incubation time accompanied with several washing steps, effectively increasing complexity and limiting its use in a simple *in-situ* test.

We describe a real-time, wash- and label-free sensor for PNA-DNA hybridization detection where the PNA probe is immobilized to the surface of the sensing gate. The motivation behind insisting wash- and label-free detection is the minimal complexity as well as significantly faster detection compared to techniques using labeling and washing steps.  We consider these as essential requirements for a practical application. We used PNA probes to decrease negative charge repulsion which is present between two DNA strands allowing efficient hybridization in low salt concentration, thus decreasing the counter-ion screening. The detection is achieved using simple discrete commercial transistor as the transducing element and the device is disposable. The used technologies allow mass production as well as scalability into array configurations. We do not aim for extremely low detection limit, but rather to robustly discriminating between specific and non-specific DNA targets.

## Background

**Figure 1:**
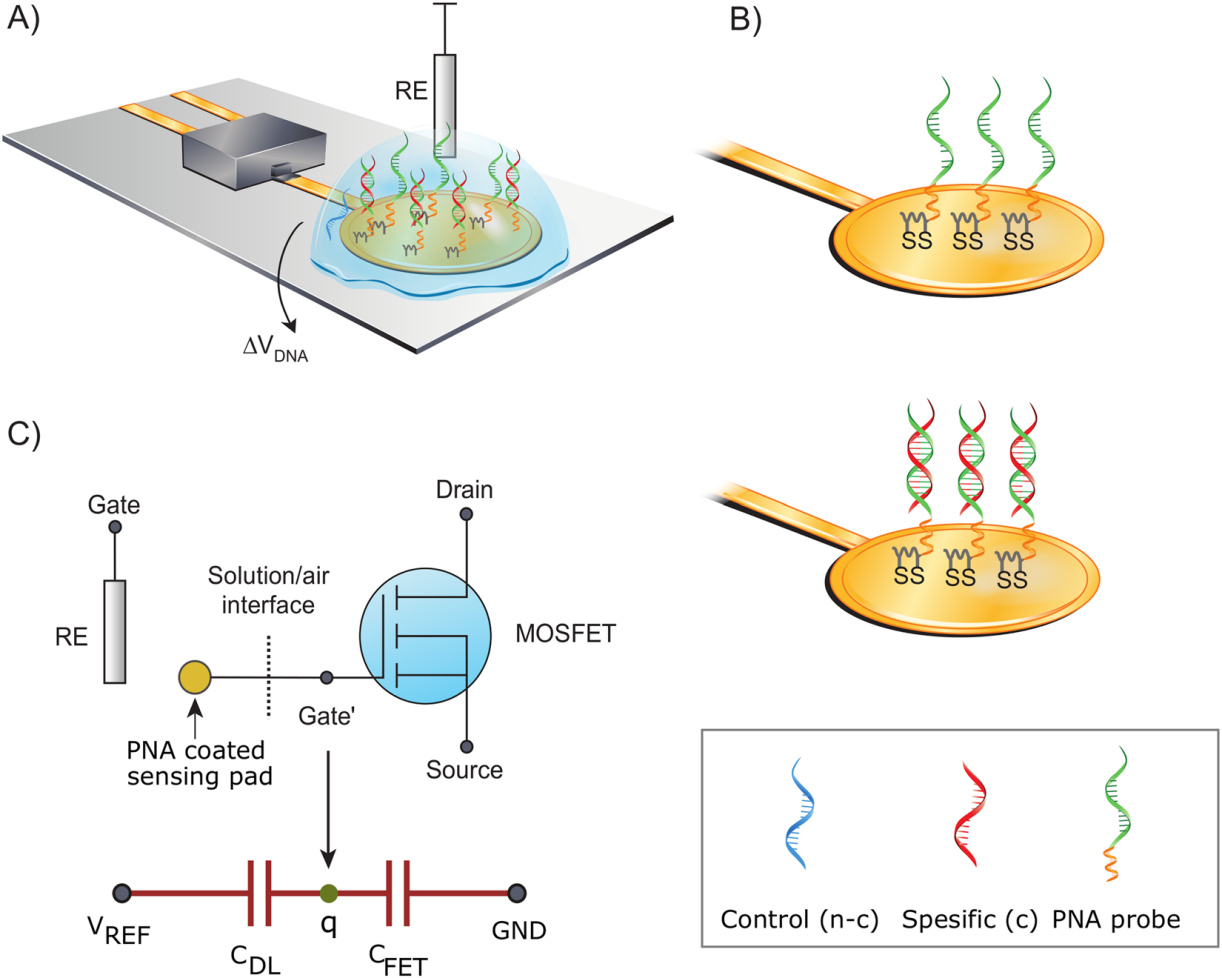
(**A**) Schematic of the DNA sensor using PNA probe (green) monolayer on gold. The gold pad is the extended-gate and it is coupled to the gate of a discrete MOSFET. (**B**) Illustrates the probe configuration with and without the hybridized complementary target. In the box the used PNA and DNA strands are shown (i) control (denoted as n-c, blue), (ii) specific target (denoted as c, red), (iii) PNA probe (green) and its linker (orange). (**C**) Equivalent circuit and a simplified capacitive model of the sensor.

The developed extended-gate DNA-FET sensor has a commercial discrete transistor as a transducing element. The gate of this MOSFET is extended with gold trace to the sensing pad^23^. This gold pad was functionalized with PNA probes. The illustration is shown in Fig. 1. The threshold voltage of the sensor (seen from the reference electrode) is given as

1 $${V}_{th}^{EGFET}={V}_{th}^{mosfet}+{V}_{cell}$$

The MOSFET threshold voltage ${(V}_{{th}}^{{mosfet}})$ remains as such without modifications as we simply connect the electrochemical cell $({V}_{{cell}}$) in series with it. The electrochemical cell in turn consists of several phases and interfaces and across each of them we have a potential difference. The sum of these potentials is the potential of the electrochemical cell^24^. Our cell consists of PNA functionalized sensing electrode, sample solution, and the reference electrode (REF), which create several constant interfacial potentials that we lump into a single constant called the standard potential ${V}_{{cell}}^{0}$. The cell potential is expressed as

2 $${V}_{cell}={V}_{cell}^{0}+{V}_{DNA}$$

In an ideal case the ${V}_{DNA}$ is the only variable of the system and by using a source-follower configuration the potential change resulting from the chemical reaction is approximately in direct relation with the observed sensor output, $\Delta {V}_{out}\approx \Delta {V}_{DNA}$.

The circuit presentation (Fig. 1C) of the sensor implies that there is no capacitive division at input of the sensor which degrade the signal transduction^21^. The used gate extension circumvents the encapsulation problem and preserves the ability to directly transduce the signal without capacitive losses. The potential shift $\Delta {V}_{{DNA}}$ occurring at the FET gate (denoted as Gate’) in the figure can be expressed as

3 $$\Delta {V}_{DNA}=\frac{\Delta q}{{C}_{FET}+{C}_{DL}}$$

where q is the DNA related charge on the surface and the two capacitances seen by this charge on its both sides are the transistor capacitance *C*_*FET*_ and double layer capacitance *C*_*DL*_, respectively.

We used PNA probes immobilized on the gold surface as a receptor for DNA target capture. PNA is a DNA analogue where the deoxyribose phosphate backbone of DNA is replaced by a synthetic peptide backbone^25^. The PNA is charge neutral and PNA-DNA hybridization lacks electrostatic repulsion found between negatively charged DNA strands and therefore, PNA-DNA hybridization is stronger than DNA-DNA binding^25,26^. The motivation behind using PNA probes stems from the higher salt concentration requirement of DNA-DNA hybridization compared to PNA-DNA hybridization in solid phase^27,28^. Thus, for DNA-DNA detection the effect of counter-ion screening is more prominent and leads to difficulty in detecting the DNA-DNA solid-phase hybridization using intrinsic charge based sensors when real-time measurements are used without washing steps. On contrary to DNA-DNA hybridization, it has been found that PNA-DNA hybridization is only weakly salt dependent and that efficient hybridization occurs in the solid phase even in very low salt concentrations^29,30^. The use of low salt has clear benefit in wash- and label free sensing since it reduces the counter-ion screening, which is considered as a significant factor limiting intrinsic charge based sensing with field-effect devices^12,31,32^.

## Results

### Fluorescence imaging of the PNA-DNA hybridization

**Figure 2:**
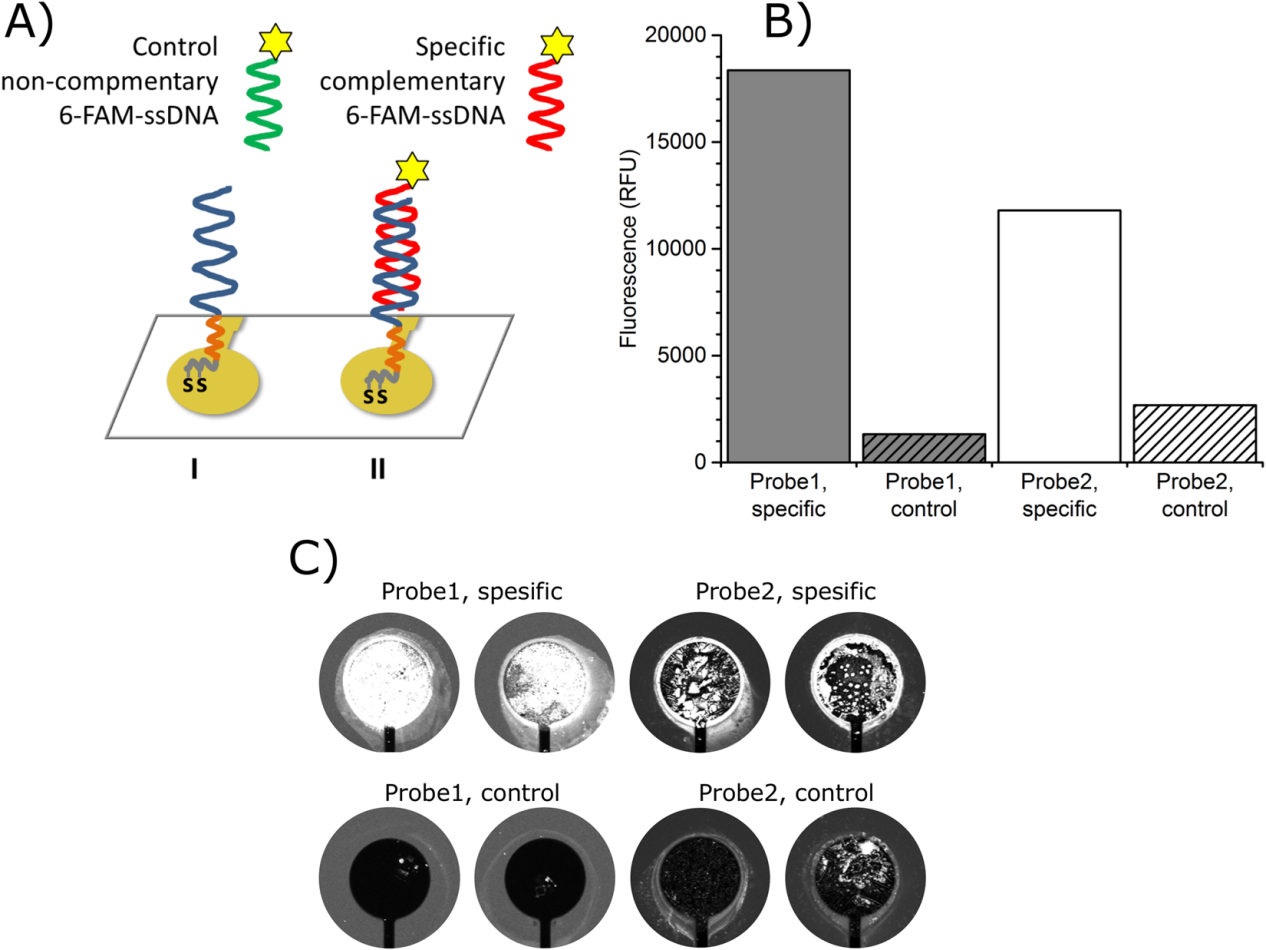
Fluorescence imaging of the FET-DNA sensor surface. (**A**) PNA probe (blue) with a spacer (orange) immobilized on gold electrode using thiotic acid (gray). (**B**) and (**C**) PNA probe1 (6.8 µM) with TTTT-spacer (gray) or PNA probe2 (1.5 µM) with 8-Amino-3,6-dioxaoctanoic acid spacer (white) on gold electrode surface was hybridized with complementary 6-FAM-ssDNA (specific) or with non-complementary 6-FAM-ssDNA (control).

Immobilization of the biomolecules including oligonucleotide probes to the gold surface via thiol-gold interaction is important and commonly used method^33^. However, total understanding of the sulfur-gold interaction has not been achieved and studies towards better control of the atomic interaction are still ongoing^34–36^. We analyzed the PNA probe surface by using fluorophore labeled complementary ssDNA in hybridization assay to ensure that the surface of the FET gate is active for hybridization. Fluorescence signals from hybridization testing are shown in Fig. 2.

Fluorescence imaging of the FET-DNA sensor electrode surface shows significant fluorescence signal difference between PNA probes hybridized with 6-FAM labeled complementary ssDNA and non-complementary ssDNA. FET-DNA sensor coated with Probe1 (6.8 µM immobilization concentration) containing TTTT-spacer between the thiotic acid and probe sequence yielded signal-to-background value of 14 (background signal from non-complementary 6-FAM-ssDA hybridization). Hybridization of PNA Probe2 with non-complementary 6-FAM-ssDNA (1.5 µM immobilization concentration) containing 8-Amino-3,6-dioxaoctanoic acid spacer yielded signal-to background ratio of 4. The results of the fluorescence measurements are shown in Fig. 2.

### Real-time PNA-DNA hybridization detection

All PNA-DNA hybridization measurements were performed by recording the sensor output in real-time. Neither washing steps nor reaction buffer changes were employed at any point during the measurements. At first, the electrodes were placed in a buffer solution for approximately 20 min in order to stabilize their potential. Next, the complementary targets were introduced to the sensing surface of the electrodes that were coated with PNA Probe1 or PNA Probe2. Three different target concentrations were added consecutively with 10 min intervals. The final ssDNA concentrations were 10 nM, 100 nM and 1 µM in the analyzed solution.

**Figure 3:**
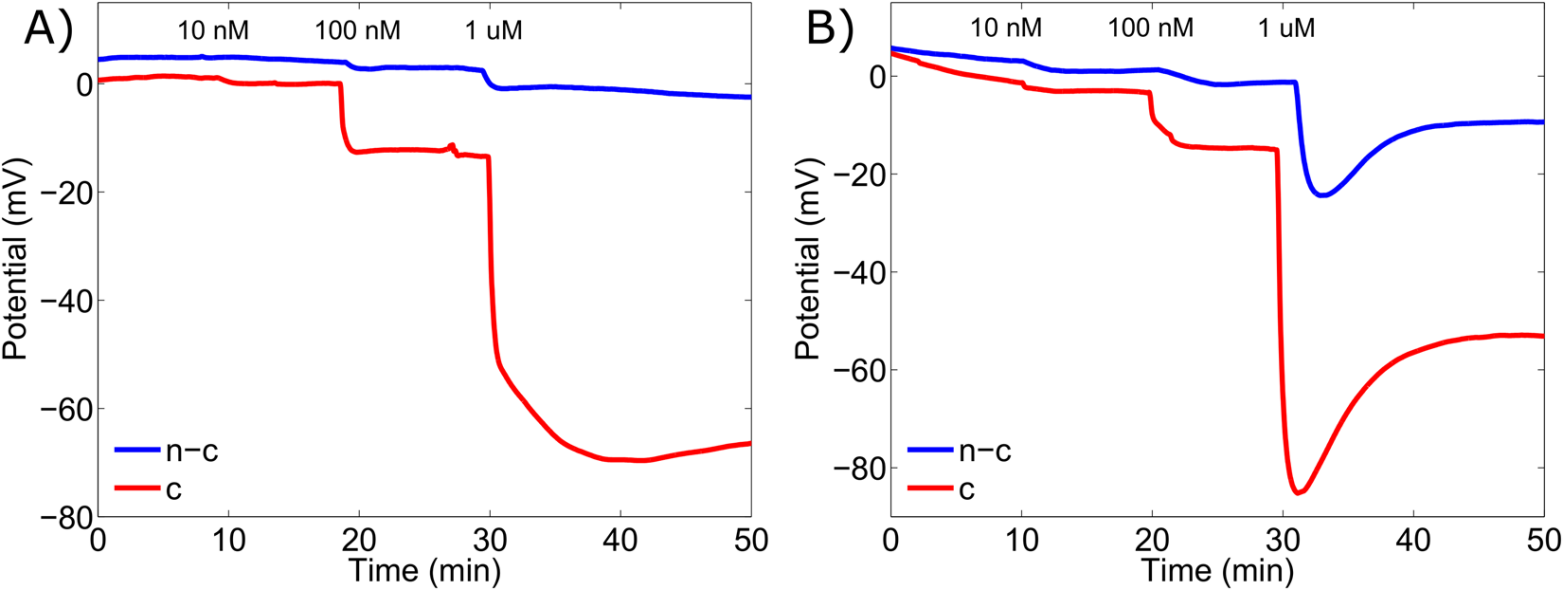
Real-time detection with field-effect transistor (FET) DNA sensor. A complementary (denoted as c) ssDNA target sequence (red) or non-complementary (denoted as n-c) ssDNA control sequence (blue) was added subsequently yielding three different concentration of 10 nm, 100 nm and 1 µM. Time points of addition are indicated in the figure. A typical response of the FET sensor containing Probe1 and Probe2 to the addition of the complementary and non-complementary targets are shown in (**A** and **B**), respectively.

All additions of ssDNA with different concentrations yielded clearly distinguishable potential shifts. The aim of this study, however, was to study the sensor specificity rather than sensitivity and for each complementary ssDNA measurement a non-complementary measurement was carried out. Typical real-time DNA detection responses are shown in Fig. 3.

**Figure 4:**
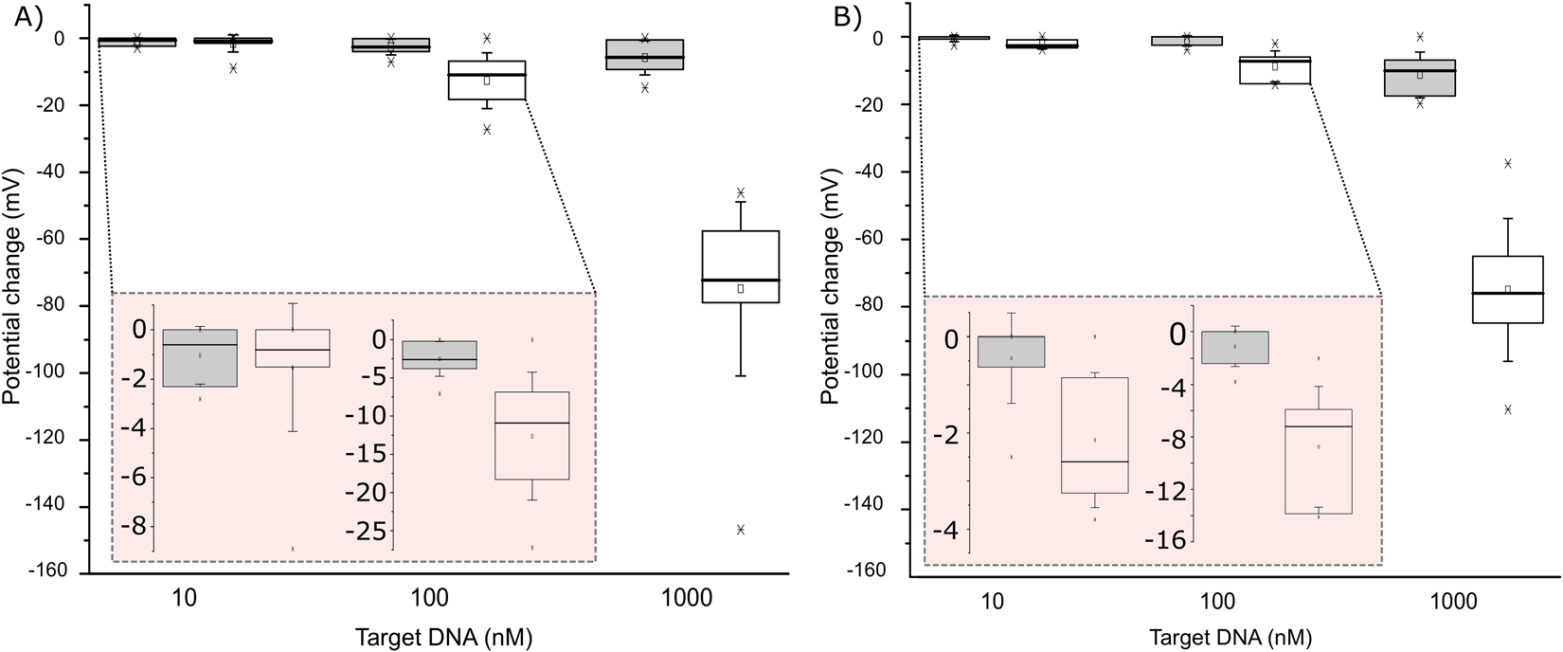
Label- and wash-free DNA detection by FET-DNA sensor. The FET extended gate gold pad surface was coated with (**A**) PNA Probe1 or (**B**) PNA probe2. Complementary ssDNA target (red) or non-complementary ssDNA (blue) was added to reaction solution in 10 min intervals. The box presents interquartile range 25–75%, and the whiskers determine the standard deviation of the results. The circles and horizontal lines in the boxes denote mean and median, respectively, and, minimum and maximum are denoted by the x-marks. Potential changes with 10 nM and 100 nM ssDNA are zoomed in the inset.

The performance of the FET-DNA sensor, in each case, was replicated in 7 to 8 parallel reactions in order to achieve statistically reliable results. The median potential shift for 1 µM complementary target ssDNA was over 70 mV with both Probe1 and Probe2 on their gate surface and, 11.9 mV and 6.4 mV, respectively, when 100 nM target ssDNA was added to the analyzed solution. The median values for 10 nM complementary target ssDNA yielded 0.8 mV and 2.6 mV for Probe1 and Probe2, respectively. The results are shown in Fig. 4 and in Table 1.

**Table 1:** Summary of FET-DNA sensor obtained median potential changes with complementary and non-complementary target ssDNA and, a statistical analysis.

	10 nM ssDNA target			100 nM ssDNA target			1 µM ssDNA target		
	c	n-c	*P*	c	n-c	*P*	c	n-c	*P*
Probe 1	0.8 mV (n = 7)	0.2 mV (n=7)	0.86	11.9 mV (n=8)	2.6 mV (n=8)	7.9 × 10^–4^	72.4 mV (n = 8)	4.2 mV (n = 8)	5.5 × 10^–5^
Probe 2	2.6 mV (n = 8)	0.0 mV (n = 7)	0.01	6.4 mV (n = 8)	0.0 mV (n = 7)	2.6.3 × 10^–3^	75.5 mV (n = 8)	9.9 mV (n = 7)	1.7 × 10^–3^

c = complementary target ssDNA.

n-c = non-complementary ssDNA.

*P* = Wilcoxon rank sum test *p*-value.

Statistical analysis of the results was performed using the two-sided Wilcoxon rank sum test that tests whether the control and target signal samples are from continuous distributions with equal median. Statistically significant difference was found between the target and control responses in all cases with the exception of Probe 1 with 10 nM ssDNA. Summary of the results with statistical analysis is presented in Table 1.

The study of the response time revealed that the response time of the sensor was very rapid and commonly 90% of the total potential change was reached within 1 min indicating the possibility of creating specific and rapid DNA sensors outside laboratory use.

## Discussion

A widely accepted reasoning for the limited performance and difficulty in detecting large macromolecules by their intrinsic charge lies in the screening effect. Ions in the solution effectively form a cloud of opposite charge around the detectable molecule. The detecting surface is in a distance from this charge complex and the effective charge detected by the sensor is greatly reduced when this distance increases. The effect can be described using the Debye length which is given by

4 $${\lambda }_{D}=\sqrt{\frac{{\epsilon }_{0}{\epsilon }_{r}kT}{\sum {e}^{2}{p}_{i}{z}_{i}^{2}}}$$

where ϵ_0_ϵ_r_ is the permittivity, k is the Boltzmann constant, T is the temperature, p is the bulk electrolyte ion number concentration, z is the ion valence and e is the elementary charge^37^. It describes the length where electrical signal has decayed to 1/e of its original value and can be described with a simple exponential relation ${e}^{-x/\lambda }$. This screening length is of a crucial importance for efficient DNA hybridization as it ensures that two negatively charged oligonucleotides can approach each other close enough for the hybridization to occur. The same effect, however, creates a clear trade-off for efficient detection capability as it screens the electric field generated by the hybridized complex. Here, we use PNA that does not carry an intrinsic charge and allows for efficient hybridization at low-ion concentrations. Using the equation presented above the computed Debye screening length is 5.1 nm with the buffer solution used in this study. An average distance between bases in DNA chain is 0.34 nm^38^ and the length of the linkers in Probe 1 and Probe2 are approximately 2 nm. If we further assume that the probes are directly standing up (orthogonal to the surface) we can make a simple conservative evaluation on the actual screening by comparing the fractions of the total charge and the amount of charge not screened by the counter-ions. This reveals that with the used configuration, the surface senses almost half of the charge of the target DNA. We believe that this is the reason for the obtained large baseline potential shifts of the sensor. Recent studies using graphene FETs support the view that increased potential shifts and sensitivity can be obtained by using PNA probes^22^ instead of a DNA probe^15^.

The linker between the probe and the surface can additionally influence the effective charge seen at the surface since the screening decay is strongest in the vicinity of the surface. Moreover, the hybridization efficiency is usually considered to decrease when approaching the surface and thus the linker length provides a trade-off between hybridization kinetics and the observable charge.

Another known performance limiting factor in extended-gate sensors with insulating sensing electrodes is the transduction effect^12^. Its impact on presented sensor construction can be considered from the simplified capacitive model. The reported input capacitance of the FET is 29 pF and by assuming the typical double layer unit capacitance of 18 µF/cm^2^ ^21,39^ with the presented electrode geometry the double layer capacitance is about four orders of magnitude larger. In the absence of capacitive division in the sensors electrical part, the capacitive division effects are negligible and it can be predicted that the electrode diameter can be reduced down 10 fold without a serious degradation in performance and clearly further if smaller input capacitance FET is used.

In this study, the addition of non-complementary ssDNA target to the analyzed solution also created a downward shift of the gate potential presumably due to non-specific adsorption that limits the sensor performance. This non-specific absorption can create both a permanent baseline shift, but additionally in some cases a significant temporary downward potential shifts were observed where the baseline returned to its value prior to the target addition. This is probably induced by hydrodynamic impact and disturbance of the double layer. The reason for different response kinetics between the probes is not fully understood, but could be due to different linkers and linker lengths. Similarly, non-spesific responses were reported for FET-DNA sensor having diamond as a gate material^21^. We have, in agreement, found that ideal responses (completely non changing) from non-specific targets are possible, but not routinely obtainable; although this might be alleviated with the use of more repeatable automated monolayer formation processes. Additionally a heat control is expected to reduce the deviation of the obtained responses by reducing the non-specific PNA-DNA binding.

Relatively high signal variation was measured especially with 1 µM specific target DNA. We expect that a major reason for the variation of the responses arose from the difference in gold sensor surface ambient air exposure time before immobilization. Organic molecules from environment absorbs on the gold because of gold surface free energy^40,41^ and can cause variance in probe coverage and density and thus, to the sensor performance. Furthermore, the PNA is poorly water soluble and therefore, the PNA immobilization concentration had variation that might had created variation on the density of the probe surface and thus, variation in electrical measurement. Controlling the above mentioned factors most likely would significantly improve the repeatability and possibly the specificity even further.

## Conclusion

We developed a low-cost FET-DNA sensor based on standard electrical components. The sensor operates in label and, notably, wash-free conditions. The sensor can routinely discriminate between complementary and non-complementary oligonucleotides in nanomolar concentrations. It is usual that intrinsic charge based measurements are not carried out in real-time, but rather are incubated for a rather long periods after which they are measured in low-ion concentration solution due to the screening effect. Here, the real-time detection is achieved with short response times. We also found that the mere sensitivity is not the performance-limiting factor, but rather the specificity. As the reaction, and thus specificity, is a surface phenomena^42^, these results suggest that using low-cost electronics does not limit the sensor performance in the presented configuration.

## Methods

### FET-DNA sensor

The gold electrodes were acquired from Genefluidic’s (CA, USA) with a Ø 2.5 mm sensing electrode. These electrodes were coupled to the gate of the discrete transistor (BSS159N, Infineon, Germany) and were coated with peptide nucleic acid (PNA) probe (see Fig. 1A)). The PNA probe sequence was designed based on *Pseudomonas aeruginosa* heat shock protein gene (*groES*). The sequence was identical to the sequence M63957 basepairs 55–66 from the GenBank of the National Center for Biotechnology Information (U.S. National Library of Medicine). The sequence is specific to *P. aeruginosa* that causes hospital acquired infections and therefore, could be used for the detection of human infections. The probe was immobilized on the gold electrode with thiotic acid that was linked on the 5′-end of the PNA Probe1 5′-thiotic_acid–TTTTCCTCTGCATGAT-3′ or Probe2 5′-thiotic_acid–8-Amino-3,6-dioxaoctanoic_acid–CCTCTGCATGAT-3′. The probes had same target binding sequence but different linkers. The PNA probes were purchased from biomers (Germany).

Probe immobilization was performed using 6 µL of 3 µM Probe1 or Probe2 (probe concentration varied from 1.5 µM to 6.55 µM because of PNA solubility issues) in immobilization buffer (10 mM Tris, pH 8, 50 mM KCl, 1.5 mM MgCl_2_) over the gold electrode overnight in humid condition at room temperature (rt). After immobilization the electrodes were rinsed with 3 ml of MQ-H_2_O and left to dry in air at rt. The surface was post-treated with 6 µL of 1 mM 6-mercapto-1-hexanol for 1 hour at rt for removing nonspecifically absorbed oligonucleotide probes from gold surface and to block the surface for decreased target oligonucleotide adsorption during the hybridization reaction^33^. Lastly the sensor surface was rinsed as described above and the sensors were used immediately.

### Fluorescence imagining of the FET-DNA sensor

The FET-DNA sensor PNA probe1 and probe2 surface was tested using fluorescence complementary oligonucleotide. 6 µL of 1 µM 6-Carboxyfluorescein (6-FAM) 5’-labeled specific complementary oligonucleotide 5′-CGATCATGCAGAGG-3′ or 6-FAM 5′-labeled non-complementary control oligonucleotide 5′-TTTATGTCGTACTAGAACCTG-3′ (purchased from Biomers) was incubated on the sensor surface for 1 h rt in reaction buffer followed by 3 mL rinsing with 10 mM Tris, pH 8, 0.6 M NaCl in order to perform fluorescence imagining of the FET-DNA sensor. Fluorescence signal from the electrode surfaces were measured by confocal laser scanning microscope using 488 nm excitation wavelength and 535/25 nm emission filter with 20 µm resolution (pixel size).

### Electrical PNA-DNA hybridization detection

Label- and wash-free electrical DNA detection was studied by stepwise additions of the complementary (or non-complementary) single-stranded DNA (ssDNA) target with increasing concentrations. 100 µL of the reaction buffer (1 mM Tris, pH 8, 1 mM MgCl_2_) was applied onto the sensor surface using adhesive film well (Genefludics) around the probe coated electrode. The target DNA solution was pipetted on the FET-DNA sensor surface and the signal was measured continuously in real-time. 1 µL of the complementary target (in reaction buffer) ssDNA 5′-CGATCATGCAGAGGA-3′ or non-complementary ssDNA 5′-TTTATGTCGTACTAGAACCTG-3′ was added into the reaction solution to achieve the final reaction concentration of 10 nM, 100 nM and 1 µM at time points 30, 40 and 50 minutes, respectively. The signals were followed continuously from the timepoint 0 min to 70 min.

### Measurement setup

We used a discrete depletion mode nMOS transistor (BSS159N, Infineon) as the transducer. We biased the transistor in a source follower configuration with a constant drain voltage of 2 V. An adhesive film well around the sensing electrode was used to confine the 100 µL droplet over the sensing electrode. This ensures that any other part of the sensor is not in contact with the solution and it additionally avoids any self-referencing issues and problems related to leakage currents. The constant bias voltage of −1.6 V to −1.8 V, depending on the transistor threshold voltage, was applied through a miniature Ag/AgCl reference electrode (RE). We measured the sensor output with a 24-bit AD-converter and post processed the data in PC. The post processing includes relating the sensor output directly to the changes at the gate potential by correcting slightly non-linear and below unity gain response of the source follower configuration. The gold surface PNA coated electrodes drift after immersing them in the buffer solution. We computationally removed this drift by subtracting the first order polynomial from the original signal. The fit is obtained from the 10 min period before the addition of the first target DNA. The baseline is shifted near zero to better reveal obtained changes in the signals. All measurements were done at room temperature and under normal lighting.
